# Bacteria-derived extracellular vesicles: endogenous roles, therapeutic potentials and their biomimetics for the treatment and prevention of sepsis

**DOI:** 10.3389/fimmu.2024.1296061

**Published:** 2024-02-13

**Authors:** Clement Yaw Effah, Xianfei Ding, Emmanuel Kwateng Drokow, Xiang Li, Ran Tong, Tongwen Sun

**Affiliations:** ^1^ Department of Critical Care Medicine, The First Affiliated Hospital of Zhengzhou University, Henan Engineering Research Center for Critical Care Medicine, Henan Key Laboratory of Critical Care Medicine, Zhengzhou, China; ^2^ Department of Emergency Medicine, The First Affiliated Hospital of Zhengzhou University, Henan Engineering Research Center for Critical Care Medicine, Henan Key Laboratory of Critical Care Medicine, Zhengzhou, China; ^3^ Zhengzhou Key Laboratory of Sepsis, Henan Sepsis Diagnosis and Treatment Center, Henan Key Laboratory of Sepsis in Health Commission, Zhengzhou, China; ^4^ Hunan Provincial Key Laboratory of Clinical Epidemiology, Department of Epidemiology and Biostatistics, Xiangya School of Public Health, Central South University, Changsha, Hunan, China

**Keywords:** bacterial extracellular vesicles, sepsis, inflammatory response, therapeutic potential, biomimetics

## Abstract

Sepsis is one of the medical conditions with a high mortality rate and lacks specific treatment despite several years of extensive research. Bacterial extracellular vesicles (bEVs) are emerging as a focal target in the pathophysiology and treatment of sepsis. Extracellular vesicles (EVs) derived from pathogenic microorganisms carry pathogenic factors such as carbohydrates, proteins, lipids, nucleic acids, and virulence factors and are regarded as “long-range weapons” to trigger an inflammatory response. In particular, the small size of bEVs can cross the blood-brain and placental barriers that are difficult for pathogens to cross, deliver pathogenic agents to host cells, activate the host immune system, and possibly accelerate the bacterial infection process and subsequent sepsis. Over the years, research into host-derived EVs has increased, leading to breakthroughs in cancer and sepsis treatments. However, related approaches to the role and use of bacterial-derived EVs are still rare in the treatment of sepsis. Herein, this review looked at the dual nature of bEVs in sepsis by highlighting their inherent functions and emphasizing their therapeutic characteristics and potential. Various biomimetics of bEVs for the treatment and prevention of sepsis have also been reviewed. Finally, the latest progress and various obstacles in the clinical application of bEVs have been highlighted.

## 1 Introduction

Sepsis is a condition that results from an overwhelming response of the body’s immune mechanisms to infection. It is characterized by severe organ dysfunction and tissue damage (1, 2) and it is a common cause of death in clinic settings. The mortality rate in hospitals is as high as 20%-30% (3, 4). Research has shown that the occurrence and development of sepsis are accompanied by inflammatory factors disorder and immune imbalance, accompanied by an infection that can arise from either gram-negative or Gram-positive bacteria (3, 5). Failure of the regulatory mechanisms in sepsis can result in excessive activation of inflammation response, which eventually leads to multiple organ dysfunction (6, 7). Currently, the management of sepsis primarily involves providing supportive care with the objective of controlling the infection through the timely administration of suitable antimicrobial agents, providing mechanical support for compromised, organs and maintaining blood pressure through fluid resuscitation and vasopressors (8–10).

Extracellular vesicles (EVs) refer to membrane vesicles that are characterized by their small size, typically ranging from nanometers to micrometers. They are naturally-produced lipid bilayer vesicles that contain membrane, extracellular, and cytosolic signaling agents. These vesicles are secreted by both eukaryotic host cells and prokaryotic pathogens during infection (11–13). EVs play a crucial role in the regulation of immune tolerance; however, it is important to note that these EVs can also elicit harmful inflammatory reactions. Numerous studies have provided evidence that the effects of EVs can be attributed to the transfer of their contents to host cells, leading to various outcomes such as apoptosis, inflammation, and increased permeability in target organs. EVs released by pathogenic bacteria (bEVs) are known to contain various pathogenic components, including proteins, nucleic acids, carbohydrates, lipids, and virulence factors. Specifically, the small nature of bEVs enables them to traverse the blood-brain and placental barriers, which are typically difficult for pathogens to cross (14). This allows bEVs to transport pathogenic agents to host cells, stimulate the host immune system, and potentially expedite the progression of bacterial infections and subsequent sepsis. bEVs stimulate the innate immune response by either directly triggering the production of immune effector molecules like reactive oxygen species (ROS) or indirectly by enhancing the synthesis of cytokines and chemokines. Leukocytes and other non-immune cells have pattern recognition receptors (PRRs) such as Toll-like receptors (TLRs). These receptors can detect and attach to pathogen-associated molecular patterns (PAMPs) and other pathogen EVs. This interaction subsequently initiates cellular signaling cascades (15). This ultimately results in the initiation of an immune response against the pathogen. Excessive release of these bEVs escalates the process of infection, increasing the host immune response, and can subsequently result in sepsis.

The distinctive attributes of EVs, including their high biocompatibility and nano-sized diameters, enable them to possess significant drug loading capacity and an extended half-life in blood circulation (16). Over the years, research into host-derived EVs has increased, leading to breakthroughs in cancer and sepsis treatments. Nevertheless, related approaches to the role and use of bacterial-derived EVs are still rare in sepsis. Herein, this review looked at the dual nature of bEVs in sepsis by highlighting their inherent functions and emphasizing their therapeutic characteristics and potential. Also, various biomimetics of bEVs such as conventional drug-coated biomimetic nanoparticles and cell-membrane-coated nanovesicles for the treatment of bEV-induced sepsis have also been reviewed in this study. Finally, the latest progress and various obstacles in the clinical application of bEVs have been highlighted.

### 1.1 bEVs and their biogenesis

**Figure 1:**
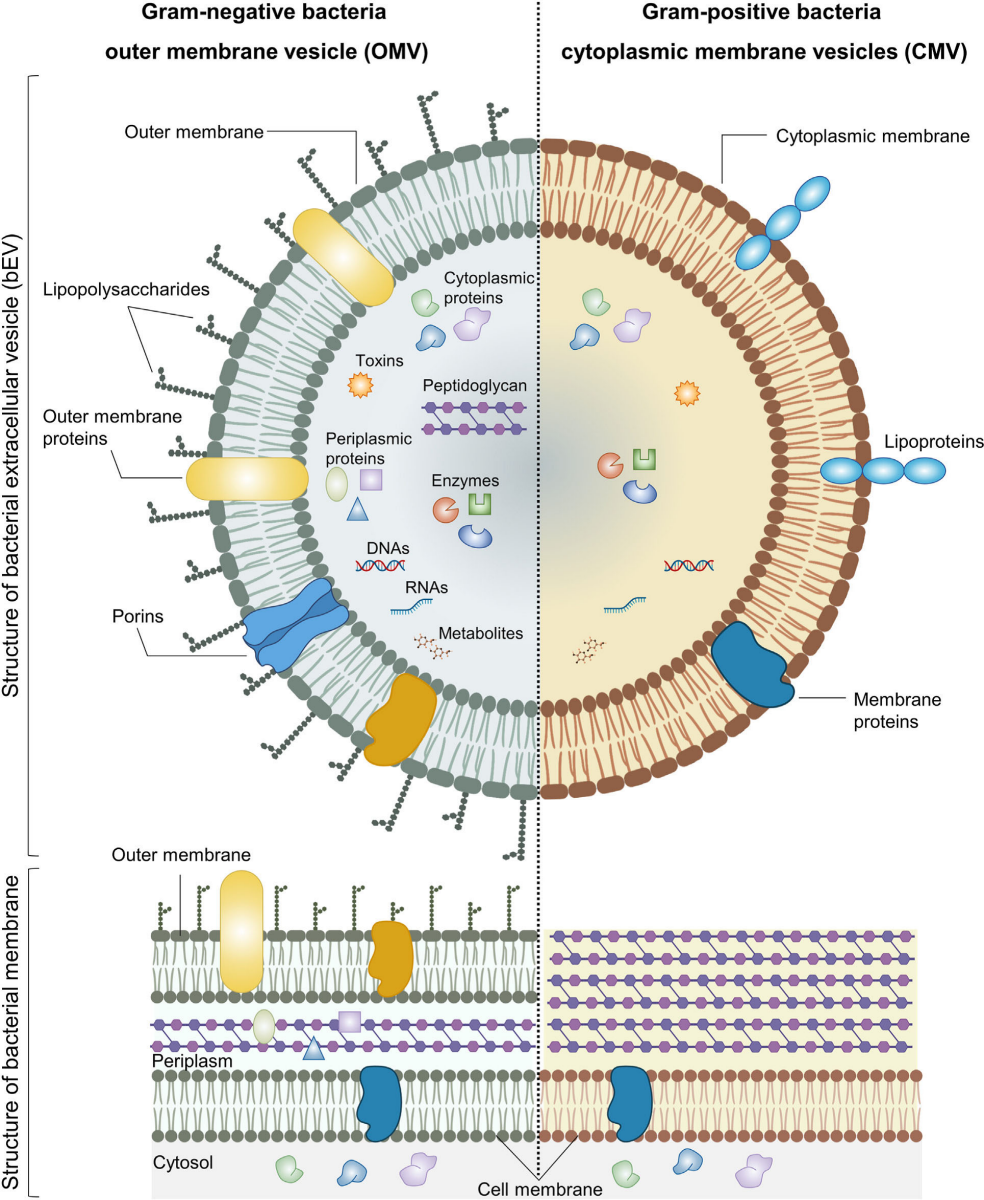
Structure and composition of bEVs secreted by Gram-negative and Gram-positive bacteria. Reproduced with permission from (28). Copyright 2022, Elsevier.

The existence of Gram-negative EVs was initially documented in 1966 (17). However, it was widely believed until recently that Gram-positive bacteria were unable to release EVs due to the substantial thickness of their cell wall (18). The production of EVs by Gram-positive bacteria, such as *Staphylococcus aureus* (19), *Streptococcus pneumoniae* (20, 21), group A Streptococcus (22) and *Bacillus anthracis* (23), has been strongly supported by various studies. Recent studies have indicated that Gram-positive EVs play a significant role in the formation of biofilms and the development of antibiotic resistance (24, 25). Additionally, these EVs have been found to transport phage and toxin receptors, thereby rendering cells more susceptible to phage-mediated attacks and other bacteria (26, 27). bEVs typically consist of a lipid bilayer membrane in which various proteins and glycoproteins are incorporated. bEVs encompass a diverse array of proteins, including enzymes, toxins, and nucleic acids (**Figure 1**). The provided description lacks specificity, making it difficult to differentiate between EVs derived from mammals and those derived from bacteria. The investigation of the composition and structure of EVs is an ongoing research area, employing diverse techniques such as electron microscopy, mass spectroscopy, and proteomic analysis. However, understanding the potential functions of bEVs presents a significant challenge at a more advanced level. bEVs are formed when Gram-negative bacteria undergo outward budding from their outer membrane, resulting in outer membrane vesicles (OMVs), or from the membrane of Gram-positive bacteria, referred to as membrane vesicles. Both types of bacterial vesicles contain nucleic acids, such as RNA and DNA cargo, as well as membrane and peri-/cytoplasmic proteins, enzymes, and toxins. OMVs display lipopolysaccharide on their surface, whereas membrane vesicles present lipoprotein (29). Similar to OMVs, EVs derived from Gram-positive cells engage in interactions with both bacterial and host cells, thereby exerting influence over diverse biological processes. Certain gram-negative bacteria with pathogenic properties, such as Shewanella vesicular M7^T^, have the ability to generate a distinct category of EVs known as inner membrane vesicles (IMVs). The formation of these vesicles occurs through the process of fission, where a protrusion of the outer and plasma membranes undergoes division. This division results in the entrapment of cytoplasmic components such as Deoxyribonucleic acid (DNA) and Adenosine triphosphate (ATP) (30). Several authors have proposed different models for the biogenesis of EVs derived from gram-negative bacteria. One of the proposed models for the origin and development of OMVs involves the disruption of the crosslinks between peptidoglycan and lipoprotein. The regulation of peptidoglycan (PG) breakdown and synthesis, as well as the formation of PG-lipoprotein (Lpp) crosslinks, is governed by a group of enzymes including PG endopeptidases. When a defect arises, the differential growth rate between the outer membrane (OM) and the underlying cell wall enables the OM to extend outward, resulting in the formation of OMVs (31). The second model that has been proposed is the aggregation of various envelope components. Bulging of OMVs is facilitated by the build-up of misfolded proteins or envelope components such as lipopolysaccharide (LPS) or PG fragments, resulting in the induction of turgor pressure (32). Another proposed model involves the enrichment of specific LPS in certain regions. Certain regions of the OM have the potential to accumulate higher concentrations of specific types of phospholipids, LPS, and/or molecules specifically associated with LPS. The molecules exhibit a tendency to protrude outward due to their unconventional structures or charges (33). The introduction of Pseudomonas quinolone signal into the outer leaflet of OM has the ability to induce curvature in the membrane and subsequently result in the formation of OMVs and this has been one of the proposed models for the biogenesis of OMVs (34). Finally, the process of downregulating the VacJ/Yrb ABC transporter has been identified as one of the proposed mechanisms involved in the formation of OMVs. The VacJ/Yrb ABC transporter facilitates the transport of phospholipids from the OM to the inner membrane. The accumulation of phospholipids in the outer leaflet of the OM can be promoted by the downregulation of this transporter, leading to vesiculation (35).

The elucidation of the biogenesis mechanisms of Gram-positive EVs remains an unresolved issue, despite the growing body of literature on the subject. EVs derived from Gram-positive bacteria are actively released into the extracellular milieu via their robust cell walls (18, 36). There exist several prevailing hypotheses that aim to elucidate the mechanisms underlying the release of EVs by Gram-positive bacteria through their cell wall. There are three potential mechanisms that may contribute to the release these EVs. First, the accumulation of EVs can lead to an increase in turgor pressure on the cell wall, resulting in their release through the plasma membrane. Second, the presence of cell wall-modifying enzymes can cause degradation of the cell wall, facilitating the release of bEVs. Thirdly, it is postulated, albeit lacking empirical evidence, that the deformation of bEVs could potentially facilitate their traversal through pores that possess a narrower width than the measured diameter of bEVs (18, 24).

## 2 Roles of bacterial EV-host interaction in sepsis

### 2.1 Etiology-overarching evidence from bacteria-derived EVs

Extracellular vesicles produced by bacteria facilitate intercellular communication between bacterial cells and their host organisms. bEVs possess the ability to modulate the immune response, initiate signaling pathways in host cells, and contribute to the development of systemic inflammation (37, 38). The mechanisms underlying the interactions between bEVs and human host cells encompass several key processes, namely the binding of bEVs to host receptors, the transfer of bEV cargo into the host cell, and the complete integration of bEVs within the cytoplasm of the host cell (39–41). Following the adhesion/binding of bEVs to host cells, three distinct pathways have been postulated for the internalization of bEVs into host cells (40, 42). These three primary mechanisms are: endocytosis, internalization through lipid rafts, and direct membrane fusion. The interplay between bEVs and host cells promotes the internalization of bEVs, the maturation of macrophages and dendritic cells, leading to the expression and release of inflammatory factors. The involvement of Toll-like receptors (TLRs) in the interactions between bEVs and host cells has been reported. Previous studies have reported that the internalization of bEVs derived from *M. catarrhalis* into human epithelial cells is facilitated through interactions with Toll-like receptor 2 (TLR2) (43). Lactobacillus and Bifidobacterium EVs were found to increase response of dendritic cell to cellular TLR2/1 and TLR4 (44). Cytokines and chemokines were released in a TLR2-dependent manner upon the interaction between Mycobacteria-derived EVs and mouse macrophages (45). TLR stimulation by bEVs leads to the activation of antimicrobial genes, and signaling of inflammatory cytokine (46, 47). Sepsis is thereby triggered when bEVs activate TLR, causing multiple organ failure and subsequent shock and death. A study has successfully demonstrated an up-regulation of TLR-2 and TLR4/myeloid differentiation factor 2 (TLR-4/MD-2) expression in hepatic and splenic macrophages of mice afflicted with experimental peritonitis induced by cecum ligation and puncture (CLP) (48). In a similar vein, Williams et al. (49) have also provided evidence showcasing a significant up-regulation of TLR-2 and TLR-4 mRNA expression in the lungs and liver of CLP mice, as compared to sham-operated mice, with this up-regulation manifesting as early as 1 hour post the onset of peritonitis. Moreover, Andonegui et al. (50) have reported that the expression of TLR-4, particularly on alveolar endothelial cells, plays a pivotal role in the recruitment of neutrophils into the lungs following LPS administration. These findings suggest that TLRs activated by bEVs may contribute to tissue injury during sepsis.

Also, the activation of immune responses through the cell peptidoglycan sensor nucleotide binding oligomerization domain containing 1 (NOD1) was observed in *Helicobacter pylori* EVs containing peptidoglycan (51). Researchers discovered that EVs from *Pseudomonas aeruginosa* caused an atypical inflammatory reaction in human monocytes via caspase-5 and activated inflammasomes in mouse macrophages. This was manifested through the formation of “spots”, the release of interleukin-1β (IL-1β), as well as cell death (52). EVs produced through *in vitro* methods by the *Staphylococcus aureus* have been observed to increase the expression of pro-inflammatory cytokines in living organisms and promote a T helper 17 (Th17) immune response (38). *In vitro* experiments have demonstrated that EVs derived from *Clostridium perfringens* exhibit an up-regulation of granulocyte colony-stimulating factor (G-CSF), IL-6, and tumor necrosis factor (TNF) (53). Also, EVs originating from *Akkermansia muciniphila* were demonstrated to decrease the production of IL-6 in colon epithelial cells (54). Furthermore, EVs derived from *Klebsiella pneumoniae* were found to induce the release of IL-1 and IL-8 by epithelial cells, as reported in a previous study (55). *Escherichia coli* was found to transmit an active heat-resistant enterotoxin (LT) to epithelial cells via EVs, resulting in the increased production of IL-6 (56).

Furthermore, bEVs have the ability to initiate a robust inflammatory cascade characterized by the release of cytokines, commonly referred to as a “cytokine storm”. There is ample evidence to support the notion that individuals diagnosed with sepsis exhibit increased concentrations of serum cytokines, which play a crucial role in the initiation of cytokine storm (57). A cytokine assay conducted through the utilization of a multiplex array in mice that possess a susceptibility to sepsis subsequent to burn injury revealed notable alterations in various cytokines. Specifically, an elevation was observed in the levels of IL-1β, IL-6, IL-17, G-CSF, granulocyte macrophage colony-stimulating factor (GM-CSF), macrophage inflammatory protein-1α (MIP-1α), RANTES (regulated upon activation, normal T cell expressed and secreted), and TNF-α (58). A comprehensive analysis was conducted to assess the plasma levels of 17 cytokines in individuals diagnosed with severe sepsis induced by bEVs. The results of this study revealed that patients experiencing septic shock exhibited notably elevated concentrations of IL-1β, IL-6, IL-7, IL-8, IL-10, IL-13, interferon‐ γ (IFN-γ), monocyte chemoattractant protein-1 (MCP-1), and TNF-α in comparison to those diagnosed with severe sepsis. Furthermore, it was observed that distinct profiles of cytokines were linked to the severity of bEV-induced sepsis, progression of organ failure, and mortality outcomes (59). Gram-negative bacteria typically elicit a host response through the release of OMVs (60). These OMVs disseminate various virulent factors throughout the host system, such as lipopolysaccharides (LPS), lipoproteins, and genetic materials. OMVs have the ability to induce pathophysiological effects in the interaction between bacteria and their host by altering the immune response of the host. Previous studies have demonstrated that OMVs can aggravate sepsis-like inflammatory response and induce the activation of cardiomyocytes, leading to sepsis-associated cardiac dysfunction (61).

The above descriptions highlight the interaction between evading bEVs, the innate immune response and sepsis. bEVs recognition and signaling are essential functions of the cells of innate immune systems and drive a coordinated immune response. One of the more intriguing aspects of sepsis is the fact that the protective and damaging host response are part of the same process, that is, the inflammatory response that is aimed to control the infectious process also underscores many of the pathophysiological events of sepsis. Toll-like receptor (TLR) or NOD acts to initiate host innate immune response to evading bEVs. This involves the activation of intracellular signal transduction pathways, leading to the production and release of proinflammatory cytokines. The excessive evasion of bEVs can trigger excessive cytokine storm which can lead to dysregulated immune response, resulting in sepsis. The atypical secretion of bEVs has the potential to disrupt the body’s immune response, thereby contributing to the development of sepsis. The administration of GW4869, a neutral sphingomyelinase inhibitor that impedes the production of EVs, demonstrated a notable enhancement in the survival rate of mice that were exposed to LPS injection or CLP. This finding implies that bEVs have a significant impact on the development of sepsis (62).

### 2.2 Etiology-overarching evidence from host-derived EVs

**Figure 2:**
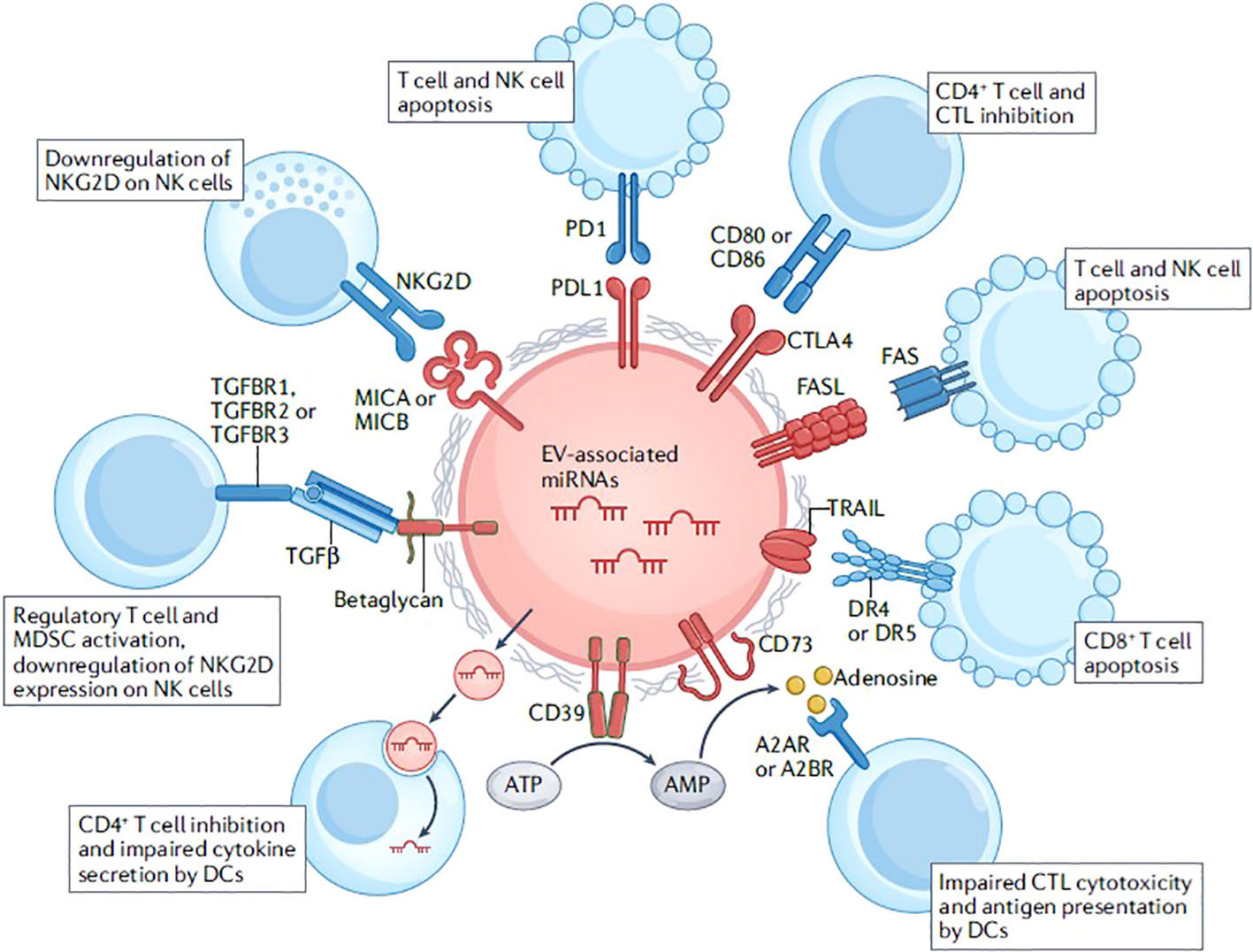
Immunoregulatory functions of extracellular vesicles. Reproduced with permission from (11). Copyright 2022, Springer Nature.

Research has also demonstrated modifications in the composition and role of EVs originating from the host during sepsis. The surface of host EVs contains immunoregulatory molecules, such as FAS ligand (FASL), programmed death ligand 1 (PDL1), TNF-related apoptosis inducing ligand (TRAIL) and cytotoxic T lymphocyte antigen 4 (CTLA4). These molecules interact with specific ligands and receptors expressed by T cells and natural killer (NK) cells, resulting in the inhibition of their activity or the induction of apoptosis. The ectoenzymes CD39 and CD73 are responsible for the conversion of ATP into adenosine, leading to the inhibition of cytotoxic T lymphocyte (CTL) responses and antigen presentation by dendritic cells (DCs). EVs derived from regulatory T cells are found to contain microRNAs (miRNAs). These miRNAs, including miR-155, Let7b, Let7d, miR-150-5p, and miR-142-3p, have been observed to exert suppressive effects on CD4^+^ T cell responses and regulate cytokine production by DCs. The cytokine transforming growth factor-β (TGFβ), known for its immunosuppressive properties, binds to betaglycan located on the surface of EVs. This interaction leads to the activation of regulatory T cells and myeloid derived suppressor cells (MDSCs), while simultaneously reducing the expression of the activating receptor natural-killer group 2, member D (NKG2D) on natural killer (NK) cells. EVs transport major histocompatibility complex (MHC) class I chain-related protein A (MICA) and MHC class I chain-related protein B (MICB) molecules, which serve as ligands for NKG2D, and can consequently result in the downregulation of NKG2D on NK cells (11) (**Figure 2**).

Due to their high abundance in the bloodstream and their heightened sensitivity to environmental changes, platelets often engage in direct interactions with invading bEVs prior to the initiation of the innate immune response (63, 64). The invading bEVs elicits platelet activation, thereby contributing significantly to the intricate pathogenesis of sepsis. The activation of platelets has been observed to result in the occurrence of disseminated intravascular coagulation (DIC) and the formation of clots within the microvasculature. These clots have the potential to induce cell death and impair the normal functioning of organs (65, 66). Platelets have been observed to gather in the microvasculature of various failing apoptotic end organs in sepsis, including the lungs, liver, and spleen. This accumulation hinders the proper circulation of blood and impairs the drainage to and from these organs (67–70). Platelets that have undergone activation due to LPS in sepsis exhibit elevated levels of transcript-1 (TLT-1) receptor (68). The TLT-1 protein has been demonstrated to enhance the process of platelet aggregation, thereby increasing the formation of blood clots and promoting the development of DIC. The interactions between platelets and neutrophils are also a contributing factor to the development of complications in sepsis. Platelets that are activated by bEVs induce the activation of neutrophils, leading to the release of neutrophil extracellular traps (NETs) (71). The process described above has been found to improve the elimination of bacteria, thereby providing advantages to the host. However, it is important to note that the formation of NETs can also result in damage to host endothelial cells. *In vivo* studies have indicated that this process may contribute to liver damage (72–75). Furthermore, it has been observed that neutrophil-platelet aggregates have the ability to release thromboxane A2 (TXA2), which is a metabolite of arachidonic acid. This TXA2 molecule is known to activate endothelial cells through the activation of G protein-coupled thromboxane receptors. The activation of receptors results in the aggregation of platelets, the activation of integrins, and an elevation in vascular permeability (76, 77). The involvement of TXA2 in activating the pulmonary vascular endothelium has been established as a crucial factor in the pathogenesis of sepsis-associated acute lung injury (ALI) (77). Inhibition of platelet-neutrophil aggregation through the blockade of P-selectin not only results in a reduction of circulating levels of TXA2, but also demonstrates improved gas exchange and increased survival in a murine model of ALI induced by sepsis (77). Platelets, despite lacking a nucleus, possess a substantial reservoir of messenger RNA (mRNA) and have the ability to modulate the translation of their transcriptome in response to external activating conditions, such as sepsis (78). The differential expression of genes associated with cell adhesion, chemotaxis, and inflammatory and immune response has been observed in septic platelets (79). In addition, it has been observed that exosomes derived from platelets of individuals with sepsis have the ability to initiate programmed cell death in endothelial cells and vascular smooth muscle cells. This effect is attributed to the heightened activity of nicotinamide adenine dinucleotide phosphate (NADPH) and the generation of reactive oxygen species, including superoxide, nitric oxide, and peroxynitrite (80).

Neutrophils are considered crucial leukocytes in the initial immune response in sepsis (81–83). Neutrophils undergo migration from the bloodstream to sites of infection in response to inflammatory signals. Once at the infection sites, they employ various mechanisms such as degranulation, phagocytosis, and the release of NETs to eliminate pathogens. Neutrophils have traditionally been acknowledged as the primary effector cells in the context of sepsis. The correlation between tissue damage in sepsis and the release of proteolytic enzymes, oxygen radicals, and NETs by neutrophils has been demonstrated. The concentration of neutrophil-derived EVs exhibited a significant increase in the bloodstream of mice that underwent CLP, a widely employed animal model for studying sepsis (84). Neutrophilic EVs have been implicated in the initiation of oxidative stress, myocardial dysfunction, and apoptosis of vascular cells (85). Furthermore, it has been demonstrated that neutrophil-derived EVs play a role in the development of septic encephalopathy through direct mechanisms and by inducing systemic immune dysfunction as a result of the disruption of neuroendocrine immune networks (85). Also, it has been observed that host EVs derived from neutrophils have the potential to play a role in the development of chronic liver dysfunction following sepsis (86).

The potential impact of EVs in sepsis extends beyond their capacity to induce harm to host cells and organs. It is worth noting that a number of host defense mechanisms related to EVs have been recently identified in experimental models of sepsis. Multiple studies have demonstrated the antibacterial effects of EVs derived from granulocytes (87). These EVs have also been found to facilitate the production of inflammatory mediators and provide protection against vascular dysfunction (88). Similarly, it has been demonstrated that EVs containing alpha-2-macroglobulin, which are secreted by neutrophilic granulocytes, play a role in bacterial elimination and inflammation reduction in a murine sepsis model (89). The acute systemic inflammatory response in sepsis was mitigated by immature dendritic cell-derived EVs through the enhancement of apoptotic cell clearance in septic rats. In a broader sense, it can be observed that EVs play a dual role in the context of sepsis, exhibiting both protective and detrimental effects. However, the specific impact of EVs is contingent upon their cell type of origin and the cargo they carry (90).

## 3 Clinical potential of bEVs in sepsis

bEVs have numerous clinical potentials. In biomedical research, they have been explored as biomarkers, vaccines, immunotherapeutic agents, and as nanodrug delivery system. EVs generated by diverse bacterial species exhibit distinct characteristics and possess the ability to selectively target various tissues or organs, facilitating the controlled release of the active substances loaded on them. These EVs hold promising prospects for therapeutic interventions in the treatment of infections and other pathological conditions.

### 3.1 Techniques for the isolation and purification of bEVs

In order to achieve the clinical potential of bEVs, there is the need to isolate and purify high quality bEVs. Many techniques including precipitation, ultracentrifugation (UC), size-exclusion chromatography (SEC), ultrafiltration (UF), density gradient centrifugation (DGC), affinity isolation, and state-of the-arts microfluidic techniques have been developed and used to isolate and purify bEVs. The isolation of bEVs from biofluids is a challenging task due to the complex nature and inherent heterogeneity of biofluids. In such situations, it will be crucial to employ a combination of purification methods that are based on complementary principles in order to effectively eliminate interfering components such as lipoproteins and host EVs (91). Several authors (92–97) have extensively reviewed on the techniques and methods for the isolation and purification of EVs and as such, this part of the review will not elaborate on this topic. However, the performance of various conventional techniques for isolation and purification of bEVs have been summarized in **Table 1**.

**Table 1:** Performance evaluation of various conventional techniques for isolation and purification of bEVs.

Technique	Efficiency	Purity	Complexity	Scalability	Yield	Ability to isolate bEVs subpopulations
Precipitation	+++	+	+	+++	++++	–
UC	++	++	+	++	++++	–
SEC	+++	+++	+++	++	++	+
UF	++++	+	+	+++	++++	–
DGC	+	+++	++++	+	++	+
Affinity isolation	+++	++++	++	+++	+++	++

– to ++++ represent performance range in arbitrary units.

### 3.2 Potential as biomarkers

In contrast to extracellular vesicles derived from the host, the utilization of bEVs derived from biofluids has not been fully explored, partially due to the persisting methodological challenges. Nevertheless, there is a growing body of evidence indicating that alterations in the microbiome associated with diseases can potentially be observed in the levels and composition of biofluid-derived bEVs. Therefore, the existence of particular biofluid-derived bEVs, such as those found in serum, may be linked to a particular infection status. This makes bEVs a promising candidate in clinical diagnosis as biomarkers for the rapid detection of inflammation and for disease diagnosis (98). To distinguish bEVs from eukaryotic EVs, the 16S ribosomal RNA (rRNA) V3-V4 hypervariable regions are commonly employed. These regions are used for the purpose of quantification, identification, and classification of bacteria in complex samples (54). Numerous studies have demonstrated the isolation of circulating bEVs from serum. The assessment of the variety and quantity of bacterial compositions in EVs present in biological fluids have been conducted using next-generation sequencing of the V3-V4 hypervariable regions in 16S rRNA metagenomic analysis (99, 100). In the study conducted by Lee et al., EVs derived from urine samples were employed to perform 16S rRNA sequencing (101). The primary objective of this analysis was to investigate and describe the altered composition of gut microbial communities in individuals diagnosed with autism spectrum disorder. The utilization of bEVs for diagnostic purposes offers a significant advantage due to their ability to circulate and be detected in readily obtainable bodily fluids. Additionally, they may offer valuable insights into the relationship between microbiota and the overall health condition of the host (39). Previous studies have demonstrated that there are specific alterations in circulating bEVs in individuals with cirrhosis and hepatocellular carcinoma (HCC). The relative abundance of Klebsiella and Acinetobacter, was found to be increased in HCC. These bacteria are known to produce LPS, which have the ability to induce inflammatory damage specifically in the liver. Remarkably, Staphylococcus showed the most significant differences between HCC and HC (102). Meanwhile, a study conducted by Kim et al. (103) examined bEVs obtained from serum to capture the diversity of ovarian cancer and track real-time tumor changes. Plasma bEVs have been utilized in the analysis of microbiome composition patterns, showing promise as a potential diagnostic marker for a range of diseases including biliary tract cancer (104) and inflammatory bowel disease (105).

Due to lack of characterization of bEVs, bEVs are not well differentiated from host-derived eukaryotic EVs. The overlapping size range (30–150 nm) of bEVs and host-derived eukaryotic EVs limits the utilization of bEVs as diagnostic biomarkers.The utilization of bEVs as a diagnostic marker is expected to continue advancing in the foreseeable future. The utilization of bEVs as a biomarker is currently in the proof of concept stage. However, its future trajectory has the potential to not only revolutionize current diagnostic mechanisms but also provide insights into the causal relationship between bEVs and pathological conditions (54). Although most of the studies conducted have been focused on cancer, same approaches can be employed to elucidate the potential of bEVs as diagnostic biomarkers for sepsis and sepsis-associated conditions. Research on the development of bEVs as a biomarker in biofluids such as saliva, serum, plasma, urine, stool and nasal secretion should be explored.

### 3.3 Therapeutic potential

#### 3.3.1 Nano-drug/biologic delivery system

Due to their capacity to encapsulate and transport diverse bioactive molecules, bEVs hold immense promise as a new form of drug delivery vehicle (106). EVs do not only improve the uptake of drugs, but they also safeguard their cargo from degradation, thereby ensuring their delivery to target cells in a functional state (107). bEVs have the capacity to transport a diverse array of drugs, encompassing radiotherapy drugs, small molecule chemotherapeutic drugs, and drugs with diagnostic implications such as peptides, proteins, and nucleic acids (28). The encapsulation of bioactive compounds within bEVs can be achieved through either *in vivo* or *in vitro* methods. Compounds of interest can be encapsulated *in vivo* during EV biogenesis. In this particular scenario, it is observed that during the process of EV biogenesis, pharmaceutical substances present within the intracellular compartment can be enclosed within the vesicles that are formed, resulting in the release of EVs containing the specific compound of interest. Allan and Beveridge (108) employed this methodology to generate *Pseudomonas aeruginosa*-EVs encapsulated with gentamicin which can deliver drugs to the target, *Burkholderia cepacia*.

In an alternative approach, purified EVs have the potential to be loaded with various types of compounds *in vitro* using electroporation techniques. The aforementioned methodology was effectively utilized to administer EVs with small interfering RNA (siRNA) (109) or with gold nanoparticles (110). Recent research has indicated that OMVs possess considerable promise in facilitating the delivery of small molecule drugs. The efficiency of electroporation as a means of loading drugs into the lumen of OMVs has been demonstrated. Passive diffusion technique can be employed for small molecule drugs that possess hydrophobic characteristics, positive charge, and can easily interact with lipophilic membranes. In the case of hydrophilic molecules, the presence of a bilayer lipid membrane may pose a challenge, necessitating the use of electroporation as the preferred method (111). The electrotransport method, known for its convenience and ease of implementation, has found extensive application in the loading of small molecular substances (112). In recent times, researchers have endeavored to employ bioengineering techniques in order to effectively load pharmaceutical substances onto OMVs. Researchers have utilized OMVs derived from non-pathogenic commensal bacteria as a vehicle for the administration of keratinocyte growth factor 2 (KGF-2), a small molecule that shows promise in the treatment of inflammatory bowel disease (113). Carvalho et al. (114) conducted an engineering experiment on *Bacteroides thetaiotaomicron* (Bt), a Gram-negative bacterium that is widely present in the intestinal microbiota of various animal species. The objective of their study was to introduce proteins derived from bacteria, viruses, and humans into the OMVs of Bt. Subsequently, the researchers employed the modified Bt OMVs as a means of transporting these proteins to the respiratory and gastrointestinal (GI) tracts, with the aim of safeguarding against infection, tissue inflammation, and injury. Their findings demonstrated that OMVs have the capability to produce the human therapeutic protein, keratinocyte growth factor-2 (KGF-2), in a stable form. When administered orally, it was found to effectively mitigate the severity of the disease and facilitate the restoration and healing of the intestinal epithelial tissue.

Although using bEVs as drug delivery system has advantages such as good bioavailability and biocompatibility, possible to express and package multiple functional components, high scalability, and the possibility of using cellular processes for drug loading and surface modifications, there are still some issues that needs more attention. These include issue of heterogeneity, nonspecific effects of natural bEVs cargoes, immunogenicity, low efficiency to load exogenous drugs and lack of controlled release mechanism.

### 3.4 Potential for vaccines

Importantly, bEVs can also serve as a valuable resource for vaccine formulations. One notable benefit associated with the utilization of bEVs for vaccination purposes is their capacity to concurrently display multiple antigens in their natural conformation, thereby eliciting robust immune responses. The utilization of OMVs has emerged as a highly promising platform for the development of vaccines. These vesicles possess a size that is most favorable for internalization by immune cells and contain molecules that activate TLRs, such as LPS which can induce an innate immune response. The utilization of bacterial vesicle-based vaccines offers several potential advantages in terms of manufacturing simplicity and cost-effectiveness. These vaccines have the capacity to represent multiple antigenic molecules, thereby mitigating the risk of escape variants. Additionally, the surface-expressed antigens within these vesicles exhibit a natural orientation, enhancing their efficacy. Furthermore, bacterial vesicle-based vaccines demonstrate stability and can be genetically engineered, further expanding their potential applications. By integrating bioengineering techniques with specific production processes, it has become possible to generate a substantial quantity of OMV particle vaccine products that are characterized by their well-defined nature, stability, and uniformity (115). Currently, a diverse range of bacterial OMVs have been employed to produce vaccines. These OMVs originate from bacteria such as *Neisseria* spp., *Helicobacter pylori*, *E. coli*, Shigella, *P. aeruginosa*, *Campylobacter jejuni*, Salmonella, *Borrelia burgdorferi*, and Vibrio (112, 116). An additional illustration of the potential therapeutic applications of bEVs involves the utilization of EVs derived from probiotics, such as *Lactobacillus plantarum* WCFS1, *Clostridium butyricum* and *Bifidobacterium longum*. The EVs derived from probiotics have the ability to activate innate immune cells, leading to the production of TNF and IL-6. This finding implies that probiotic EVs could potentially be utilized as innovative vaccine adjuvants. The ability of EVs produced from gram-negative bacteria to stimulate antigen-specific CD8^+^ T-cell responses has been documented (117), and this is crucial for immunization against intracellular bacteria, viruses, and sepsis. **Table 2** provides a summary of the different vaccines and adjuvants based on bEVs that are currently in clinical and preclinical stages of development.

**Table 2:** Various bacterial EV-derived vaccines at various clinical stages.

Bacterium	Type of OMV	Composition	Target	Stage	Ref
*A. baumannii* ATCC 19606	mdOMV	ΔlpxD mutant OMV	*A. baumannii* infection	Preclinical	(118)
*A. baumannii* ATCC17978 and LAC-4	nOMV	OMV with aluminium phosphate adjuvant	*A. baumannii* infection	Preclinical	(119)
*E. coli* F4 and F18	cOMV	OMV coated into/on NPs	ETEC infection	Preclinical	(120, 121)
*S. Typhi* BRD948	mdOMV	GMMA with heterologous *S. Typhi* Vi Ag and homologous O:2 OAg	Salmonella infection	Preclinical	(122)
*E. coli* DH10B	mdOMV	*E. coli* mutant OMV expressing HA and RBD	H1N1 and MERS-CoV infection	Preclinical	(123)
*S. sonnei* 1790	mdOMV	GMMA with OAg (1790GAHB)	*S. sonnei* infection	Phase I in Europe; Phase II in Africa	(124, 125)
*S. Typhimurium* 2189 and *S. enteritidis* 618	mdOMV	GMMA with OAg	Salmonella infection	Preclinical	(126)
MenB	nOMV	Polyhistidine triad protein D; OMV and alum as adjuvants	*S.* *pneumoniae* infection	Preclinical	(127)
	dOMV	4CMenB OMV (Bexsero)	*N. gonorrhoea* infection	Preclinical	(128)
	(md+d)OMV	ΔporAΔporB MenB OMV	MenB infection	Preclinical	(129)
*E. coli* MC4100	mdOMV	ΔnlpI mutant OMV expressing ClyA-M2e4xHet	Influenza	Preclinical	(130)
*H. pylori* 7.13	nOMV	OMV	*H. pylori* infection	Preclinical	(131, 132)
*S. aureus* RN4220, Newman, N315, Mu50, and ATCC 25923	mdCMV	Δagr CMV expressing dengue virus antigens	Dengue virus infection	Preclinical	(133)
*B. melitensis* 16M	nOMV	OMV with Poly(I:C)	*B. melitensis* infection	Preclinical	(134)
*S. aureus* S29213, BW15 and BWMR26	cOMV	OMV coating ICG-loaded magnetic mesoporous silica NPs	*S. aureus* infection	Preclinical	(135)
*N. gonorrhoea FA1090*	mdOMV	OMV with IL-12	*N. gonorrhoea* infection	Preclinical	(136)

cOMV, coated outer membrane vesicle; CMV, cytoplasmic membrane vesicle; nOMV, natural outer membrane Vesicle; dOMV, detergent-extracted outer membrane vesicle; MERS-CoV, Middle East respiratory syndrome coronavirus; RBD, receptor binding domain; ETEC, enterotoxigenic Escherichia coli; mdOMV, mutant-derived outer membrane vesicle; HA, hemagglutinin; MenB, Serogroup B Meningococcal.

One of the primary obstacles in using bEVs as vaccines lies in devising an effective approach to genetically modify the parent bacteria in order to augment the yield and their immunogenicity, while simultaneously reducing their potential reactogenicity. Furthermore, there is a growing scholarly interest in examining bEVs heterogeneity on the immunization response. The development and utilization of OMV modifications, specifically the incorporation of heterologous antigens using the generalized modules for membrane antigens (GMMA) technology and the plug-and-display technology, have been extensively studied and established as a reliable approach. The GMMA technology involves the integration of heterologous antigens into the vesicular compartment and modifies the acylation process of lipid A. This results in the generation of penta-acylated LPS with reduced endotoxicity, while still maintaining the important O antigen component of the LPS (137). The plug-and-display platform offers various plasmid-encoded polysaccharide biosynthetic pathways, which can be easily introduced into *E. coli*. This facilitates the rapid development of customized multivalent OMV-based vaccines (28). As the aforementioned alterations have a discernible impact on the intercellular communication between bacteria and cells, they consequently influence the efficacy of OMVs as vaccines. Below are some protein systems employed for the engineering of various antigens and enzymes on the surface of OMVs for the purpose of vaccine production.

**Figure 3:**
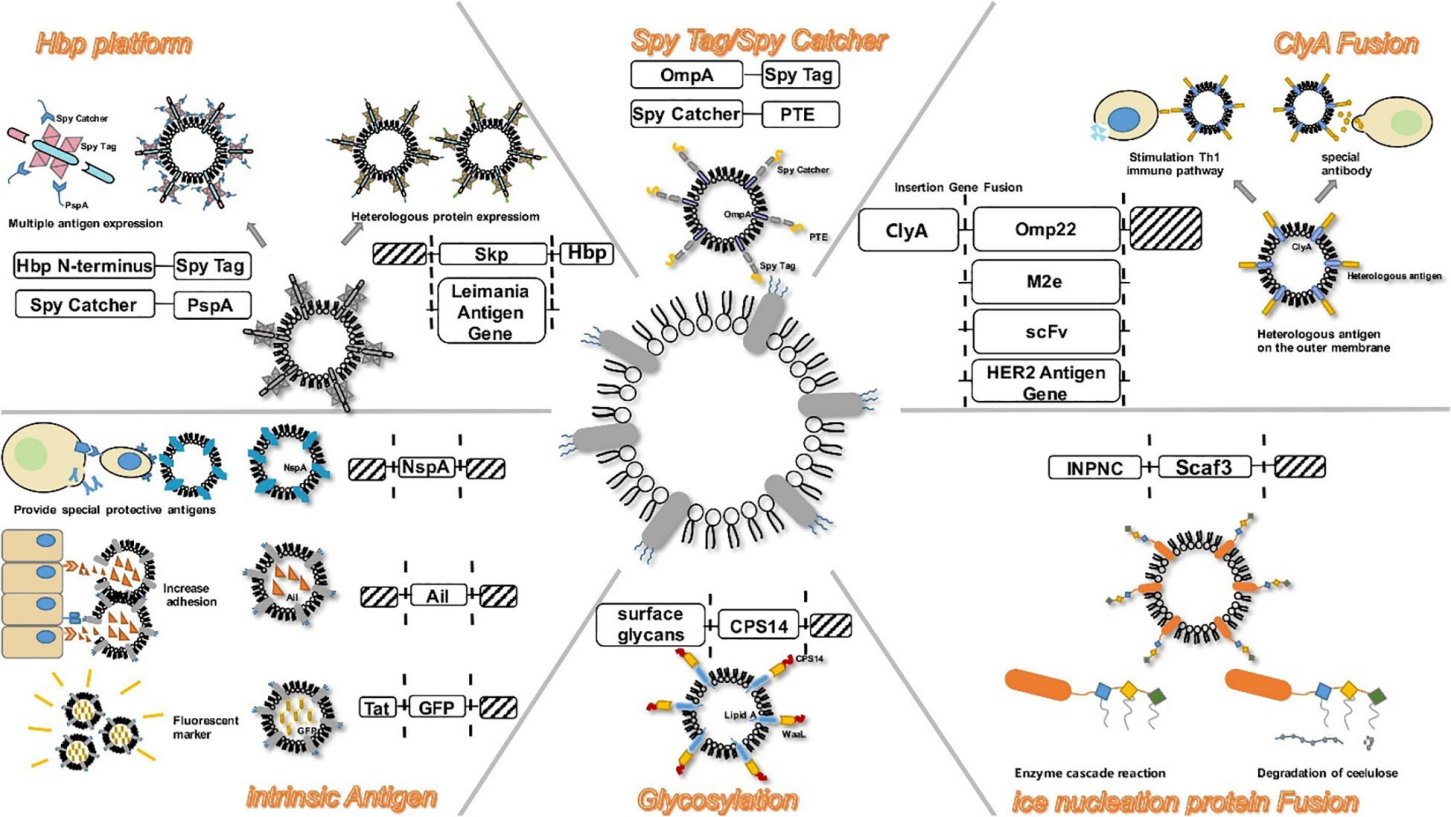
Modification strategies on OMVs during bEV vaccine production. Reproduced with permission (139). Copyright 2020, Frontiers.

The cytosolic protein, Cytosolic A (ClyA), with a molecular weight of 34-kDa is encoded by the ClyA gene located on the K-12 chromosome of bacterium *E. coli* (138). In recent times, scholars have shown a preference for the genetic fusion of ClyA (**Figure 3**). ClyA plays a crucial role in facilitating the accumulation of heterogenous proteins to specific sites where OMVs are released by acting as a signal sequence. This results in the presentation of functional proteins on the surface of OMVs (140). The gene responsible for the expression of the heterologous protein facilitates the production of a recombinant outer membrane protein through its fusion with the ClyA gene. Due to its anchorage to the outer membrane, ClyA enables the extracellular expression of heterologous antigens. Direct transport of antigens into host cells is facilitated by vesicles produced by pathogens. It has been observed that the efficiency of antigen delivery is significantly enhanced by 8-fold with the use of recombinant ClyA-OMV (141). Subsequent investigations have provided further insights into the multifaceted effects elicited by protein fusion on ClyA. Inconsistencies have been observed in the results obtained from fusion with the N-terminal of ClyA. Conversely, C-terminal fusions consistently produce proteins that retain their biological functions (140).

Secretion of Autotransport (AT), a significant virulence factor with a molecular weight typically surpassing 100 kDa is a common feature in Gram-negative bacteria. The AT system exhibits a notable degree of simplicity and is postulated to harbor all information pertaining to the inner periplasm and the extracellular translocation of protein. The utilization of the AT system in Gram-negative bacteria as a mode for the transportation for diverse heterologous fusion partners has been reported (142). In the majority of instances, variants of Neisseria immunoglobulin A (IgA) protease and endogenous *E. coli* AT AIDA-I (adhesin involved in diffuse adherence) are commonly employed to genetically manipulate antigens with a specific focus on surface presentation (143). In a recent study, the skp protein of Enterotoxigenic *Escherichia coli* (ETEC) was effectively incorporated into OMVs using AT technology. In order to develop a potentially effective vaccine, OMVs-skp protein was genetically linked with glutathione-S-transferase (GST) epitope and subsequently combined with cholera toxin (144). Studies have shown that the AT platform can effectively induce CD8^+^ T cell to respond to specific antigens through the expression of ovalbumin hemoglobin protease on the surface of OMVs that have been engineered (117). The AT system, unlike the ClyA system, demonstrates efficacy in presenting multiple antigens through a subtly distinct approach. Compared to the ClyA fusion system, the AT system offers the advantage of possessing multiple insertion sites for heterologous sequences, enabling the simultaneous binding of multiple antigens. This facilitates the development of a polyvalent recombinant live vaccine. The significance of this is substantial as multivalent vaccines play a crucial role in the prevention of infectious diseases, including tuberculosis (145).

A practical method for the alteration of anchoring proteins is the use of the Spy Tag/Spy Catcher system, a protein that has the ability to form a covalent bond with its counterpart SpyTag (146). This enables engineered OMVs to perform antigen delivery with high efficency (**Figure 3**). In a study, Park et al. (147) used an enzyme scaffold to help with protein expression within bacterial cells and a truncated ice nucleation protein anchor to immobilize the protein on the outer membrane. By using a truncated ice nucleation protein gene obtained from pPNC20, a plasmid known as pINP was created (148). This system offers an efficient means of presenting various enzymes on OMVs. The enzyme complex that has been assembled not only maintains its complete functionality, but also exhibits a significantly enhanced cellulose hydrolysis rate, surpassing that of conventional enzymes by a factor of 23. Therefore, this approach can serve as a simple platform for the effective functioning of numerous enzymes as nano-biocatalysts (147). The utilisation of diverse specific binding domains enables the design of scaffolds, thereby facilitating the presentation of a limitless array of functional proteins on OMVs.

Research has demonstrated that the plug-and-display technology offers a swift advancement in the development of personalized multivalent OMV-based vaccines. On the other hand, a separate study suggests that the GMMA technology demonstrates a greater immunization impact in mice when compared to the plug-and-display system (126). The potential discrepancy in immunogenicity may be ascribed to the possibility that GMMA could function as a more immunogenic structure to present antigens to the immune system, consequently resulting in a targeted antibody response (126).

#### 3.4.1 bEV-derived vaccines used in sepsis models

Vaccines developed from bEVs have been employed, although not extensively, for the protection against sepsis in various murine models. Kim et al. (149) have shown that immunization with EVs from *E. coli* elicit potent vaccination effects against bacterial sepsis. The administration of this OMVs produced from *E. coli* effectively avoided the development of systemic inflammatory response syndrome (SIRS) induced by OMVs. The protective mechanism of OMV immunization was shown to be predominantly mediated by the activation of T cell immunity (the activation of Th1 and Th17 cell responses), rather than B cell immunity. Specifically, the production of IFN-γ and IL-17 from T cells, in response to OMV-Ag stimulation, played a crucial role in conferring protection. Another study evaluated whether *K. pneumoniae*-derived EV vaccines have some protective effects against bacteria-induced sepsis. In a murine model, the novel vaccine prevented bacteria-induced lethality and offered protection against bacteria-induced sepsis (150). Also, Wang et al. (19) considered that *S. aureus* EVs can serve as a vaccine platform if their cytotoxicity can be abrogated, and this was accomplished by purifying EVs from an ΔagrΔspa mutant of strain JE2. Immunization with the engineered-EVs provided significant protection and the detoxified EVs that over-expressed non-toxic α-hemolysin (Hla) mutant Hla_H35L_ and LukE were immunogenic, elicited toxin neutralizing antibodies, and protected mice in a *S. aureus* lethal sepsis model, indicating that these naturally produced vesicles have potential as a novel vaccine platform. In a different murine sepsis experiment, mice were subjected to immunization with OMVs derived from a clinically isolated strain of *Acinetobacter baumannii* that exhibited resistance to multiple drugs. The administration of OMVs through intramuscular immunization elicited a prompt and robust humoral immune response, characterized by the rapid generation of elevated levels of IgG antibodies specifically targeting OMVs. In this study, it was demonstrated that both active and passive immunization strategies exhibited efficacy in protecting the mice against challenges posed by homologue bacteria (151). Also, the isolation of OMVs from *Acinetobacter baumannii* and their subsequent utilization as a vaccine in a murine model of disseminated sepsis was detailed by McConnell et al. (152). The process of immunization elicited a strong humoral immune response characterized by the generation of antigen-specific IgG and IgM against various bacterial antigens. Furthermore, it is noteworthy to mention that the process of immunization resulted in the production of both IgG1 and IgG2c subtypes. Mice that received this immunization exhibited reduced bacterial loads in their tissues and decreased serum levels of the pro-inflammatory cytokines IL-6 and IL-1β following infection, as compared to the mice in the control group.

The aforementioned studies underscore the considerable potentials of novel bEV vaccines owing to their capacity to elicit a diverse array of immune responses, thereby potentially surpassing the efficacy of conventional vaccine alternatives in certain instances. Moreover, it is worth noting that the inherent self-adjuvating properties exhibited by bEVs effectively obviate the necessity for supplementary adjuvants, thereby mitigating the potential side effects associated with their usage. Therefore, there must be an urgent focus on the development and approval of bEVs-derived vaccines that can be used for the protection against sepsis.

### 3.5 Potential as therapeutic targets

The mechanisms associated with bEVs can be strategically leveraged as potential therapeutic targets. Through the strategic manipulation of bEVs production, immune activation mechanisms of bEVs, and by disrupting the integrity of bEVs, it is possible to exert precise control on their immune modulatory roles and alleviate excessive inflammatory response, thereby preventing sepsis. Targeting bEVs production and/or their activity may present a promising therapeutic approach for the treatment of infections and associated conditions such as sepsis (153, 154).

#### 3.5.1 Inhibition of bEV virulence activity and destruction of bEV integrity

Therapeutic strategies that demonstrate the ability to impact the integrity of bEVs and/or hinder the functions of their virulence factors have shown promise. Fosfomycin and cannabidiol have been shown to possess the capability to reduce the hemolytic activity exhibited by *S. aureus* CMVs (155). Furthermore, it has been postulated that cannabidiol may potentially compromise the structural integrity of *S. aureus* CMVs, hence impeding the transportation of hemolysin to the host (155). Fosfomycin additionally demonstrates the capacity to reduce the activity of myeloperoxidase induced by CMVs of *S. aureus*, thereby potentially offering advantages in the mitigation of vascular permeability (156). Also, it has been observed that resveratrol exhibits the capacity to diminish vascular permeability that is instigated by periodontopathic bacterial vesicles. These vesicles are known to impede the production of vascular endothelial growth factor (VEGF), and this reduction in vascular permeability may be attributed to the concurrent suppression of protease activity within the vesicles (157). Moreover, it has been observed that the compound epigallocatechin gallate, which is a galloylated catechin, exhibits the ability to impede the functions of leukotoxin A which is contained in the OMVs of the periodontal pathogen, *Aggregatibacter actinomycetemcomitans*. This inhibition is achieved through the alteration of the secondary structure of the leukotoxin A and the subsequent hindrance of its interaction with cholesterol present in the membrane of the host cell (158).

#### 3.5.2 Blocking of bEV-stimulated immune activity

One of the primary catalysts for immune activation in relation to bEVs is the release of proinflammatory cytokines. These cytokines play a crucial role in instigating diverse inflammatory response that result in sepsis, extensive organ necrosis, vascular permeability, tissue damage, and potentially fatal outcomes (159). Hence, the inhibition of immune activity stimulated by bEVs emerges as a plausible therapeutic pathway for sepsis. Previous studies have demonstrated that TAK-242, a bEV-induced activity inhibitor, has the ability to target TLR4 and reduce the upregulation of IL-1β and TNF induced by *E. coli* EVs. The inhibition efficacy was 78% and 97%, respectively (160). Amelioration of systemic inflammatory response syndrome (SIRS) induced by OMV was observed through the administration of salbutamol and nortriptyline, which exhibited inhibitory effects on the release of IL-6 and tumor TNF from macrophages (161). The suppression of proinflammatory responses induced by EVs obtained from various bacterial species has been observed with the administration of budesonide, N-acetyl-L-cysteine, and fluticasone (162, 163). Moreover, it has been demonstrated that ethyl pyruvate possesses the capability to impede various signaling pathways involved in the immune responses triggered by LPS and *Escherichia coli* OMV. These pathways include the activation of the NLRP3 (nucleotide-binding domain, leucine-rich–containing family, pyrin domain–containing-3) inflammasome, the release of IL-1α and IL-1β, and the initiation of pyroptosis mediated by caspase-11 (164).

#### 3.5.3 Targeting the production and secretion of bEVs

A study has demonstrated that the suppression of the biogenesis pathways effectively hinders the production of bEVs, resulting in a notable decrease in the delivery of their harmful constituents and subsequent alleviation of sepsis (159). Based on this, various compounds have been introduced with the aim of inhibiting the production of bEVs (159). The administration of GW4869, a neutral sphingomyelinase inhibitor that impedes the production of EVs, demonstrated a notable enhancement in the survival rate of mice that were exposed to LPS injection or CLP. This finding implies that bEVs production have a significant impact on the development of sepsis and their inhibition will alleviate their effects (62). The membrane vesicles of *Pseudomonas aeruginosa* is known to deliver several virulence factors as a cargo. Therefore, inhibiting their production is a potential therapeutic target. Tashiro et al., used 4-hydroxyindole, 5-hydroxyindole, 6-hydroxyindole, isatin, and oxodole, which are bEV production inhibitors, to repress membrane vesicle production and Pseudomonas quinolone signal synthesis with inhibitory efficacies ranging from 55% to 92% (165). Peptidyl arginine deiminases (PADs) represent a group of enzymatic entities that are activated by calcium and play a pivotal role in the process of bEV production. In *Escherichia coli*, it was observed that the utilization of PAD-specific inhibitors, namely BB-Cl-amidine and GSK199, resulted in a notable decrease in OMV production, with reductions of 53.8% and 66.4%, respectively. However, in the Gram-positive bacterium *S. aureus*, a study demonstrated a reduction of 7.6% and 22.5% in CMV production (166). The observed disparities could potentially be attributed to a divergent impact of the pharmaceutical agent on the distinct biogenesis pathways of bEVs in Gram-positive and Gram-negative bacteria. Additionally, the inhibition of CMV production by *S. aureus* has been further elucidated through the introduction of a novel target, namely, sigma factor B (SigB). A compound known as Rhodomyrtone, classified as an acylphloroglucinol, has been observed to exhibit the ability to inhibit the activity of SigB and subsequently reduce the production of bEVs during the exponential growth phase. The suppression rate was up to 86.7% at a Rhodomyrtone concentration of 0.25 μg/ml (155). Additionally, a scholarly investigation has documented that cannabidiol exhibits a notable inhibitory impact on the secretion of OMVs in *E. coli*. However, it does not exhibit a similar inhibitory effect on the secretion of CMVs in *S. aureus* (167).

Based on the above descriptions, the immunomodulatory functions associated with bEVs which can result in sepsis and other inflammatory conditions, can be prevented by utilizing and targeting various biogenesis pathways, immune regulatory mechanisms and the structural integrity of bEVs through the development of new bEVs inhibitors. Also, clinical trials must be utilized to validate both the efficacies and the potential toxicity of such inhibitors as potential therapeutic options for sepsis prevention and treatment.

## 4 Biomimetics for the treatment and prevention of sepsis

### 4.1 Conventional drug-coated biomimetic of bEVs for the treatment and prevention of sepsis

In recent years, researchers have mimicked the structure and composition of bEVs and have designed various EV-mimic drug-loaded nanoparticles for the treatment and prevention of sepsis. Park et al. (168) synthesized nanovesicles (NVs) with high similarity to bEVs and conducted an investigation into the therapeutic efficacy of these NVs in treating sepsis-associated inflammation (168). The physical characteristics of the NVs were examined through the utilization of transmission electron microscopy and nano particle tracking. The findings indicated that the NVs possessed a spherical morphology, with a diameter ranging from 50 to 150 nm. Additionally, a comprehensive analysis of gene ontology, reveals that the proteins that make up the NVs were associated with various biological pathways, including inflammation, gonadotropin, and angiogenesis making these NVs a good mimic of bEVs. Then, through the injection of *E. coli* (OMV, 15 μg- intraperitoneal injection) and NVs (2* 10^9^- intraperitoneal injection), a 6h *in vivo* sepsis murine model was established. The administration of NVs resulted in a decrease in ocular secretions, partial normalization of body temperature, as well as a reduction in monocyte and neutrophil numbers in the mice. Furthermore, NVs exhibited inhibitory effects on the expression of TNF-α and IL-6. Therefore, the potential of NVs in mitigating septic inflammation and minimizing liver injury is apparent and has a promising prospect.

In addition to the vesicles generated by various bacteria, the integration of nanotechnology with bacterial mimic methods has been employed to create efficacious prophylactic platforms. Wang et al. (169) introduced innovative techniques for the artificial generation of bacterial double-layered membrane vesicles (DMVs) from bacterial sources. The researchers postulated that it is possible to harness the potential of DMVs in order to develop vaccines that can effectively mitigate the occurrence of sepsis caused by bacterial pathogens. First, a nitrogen cavitation technique was devised to effectively produce DMVs from *Pseudomonas aeruginosa*. The utilization of cryo-transmission electron microscopy (cryo-TEM) imaging techniques, in conjunction with proteomics analysis, has provided valuable insights into the structural composition of DMVs. These investigations have revealed that DMVs originate from the parent bacterial cell membrane and exhibit a diverse array of bacterial antigens, making them a very good biomimetic of bEVs. In contrast to OMVs, it has been observed that the immunizations with DMVs exhibit a greater efficacy in mitigating the occurrence of sepsis induced by *Pseudomonas aeruginosa*. In the murine *Pseudomonas aeruginosa*-induced sepsis model, it was observed that the administration of DMVs resulted in a notable improvement in mouse survival subsequent to the immunization of mice with DMVs. The increased efficacy in safeguarding against bacterial infection can be attributed to the increased adaptive immunity and distinctive biodistribution potential of DMVs. In addition, the researchers employed bioengineering techniques to manipulate *E. Coli*, with the purpose of inducing the expression of a ligand on the bacterial membrane that specifically targets tumors. The authors conducted an investigation wherein they successfully achieved the remote loading of doxorubicin into DMVs derived from RGD-engineered bacteria. The outcomes of their experimentation revealed that, in comparison to free drugs, DMVs that were loaded with drugs exhibited a notable capacity to prevent the growth of tumors. Additionally, it was noted that the concentration of drug-loaded DMVs in blood was three to four times higher than that of OMVs after 24 hours. This suggests that drug-loaded DMVs have the potential to accumulate in the spleen for an extended period, leading to enhanced immunization compared to OMVs. Also, Chen et al. (170) utilized the inherent biocompatibility and immune activation properties of Salmonella strain VNP20009 in order to generate DMVs that exhibit improved systemic safety and enhanced immune response. The study explores the photothermal effect of polydopamine when exposed to radiation. To enhance the possible synergies between photothermal therapy mediated by mesoporous polydopamine (MPD) nanoparticles and immunotherapy mediated by VNP20009-derived DMVs, the researchers coated the surface of MPD nanoparticles with the synthesized DMVs. Although the above descriptions and experiments were for cancer-targeted therapy, same approaches can be used to remotely load DMVs with anti-inflammatory drugs for sepsis targeted therapy.

Bacterial protoplast has been bioengineered to alter their structural integrity by eliminating their cell walls. This transformation is achieved through a multitude of techniques, including enzymatic degradation of the peptidoglycan layer and the strategic manipulation of cell wall synthesis proteins via genetic knockout mechanisms (171). Kim et al. (172) subjected bacterial protoplasts to a process of extrusion, resulting in the generation of nano-sized vesicles. These vesicles, which closely resemble bEVs, were obtained by harvesting them from the interface between the iodixanol layers of 10% and 50% in a two-step density gradient ultracentrifugation. The extracellular vesicles-mimicking nanovesicles, derived from bacterial protoplasts, were subsequently employed for the encapsulation of targeted antigens on the inner membrane. The nanovesicles demonstrated a notably enhanced yield and safety in comparison to extracellular vesicles obtained from the original bacterial source. Significantly, the administration of these nanovesicles resulted in enhanced immunity against bacterial sepsis during a murine experiment.

Previous studies (173, 174) have indicated that superparamagnetic iron oxide nanoparticles (SPIONs) possess comparable characteristics to bEVs, exhibit minimal cytotoxicity, and demonstrate favorable biocompatibility. By mimicking the nano-structure and the biodistribution properties of bEVs, Xu and colleagues (175) conducted a study wherein SPIONs were synthesized through the encapsulation of iron oxide with a modified polymer known as polyglucose sorbitol carboxymethyl ether. The primary objective of this research was to gain insights into the underlying mechanism through which SPIONs modulate the inflammatory response (175). In an experimental murine model of liver injury caused by LPS-induced sepsis, SPIONs exhibited a mitigating effect on the inflammatory response commonly associated with sepsis. This was evidenced by a reduction in the infiltration of inflammatory cells within the liver and an increase in the levels of IL-10 (a cytokine known for its anti-inflammatory properties), by activating autophagy in hepatic macrophages, which are the primary cellular population expressing IL-10 in the liver. In addition, the administration of SPIONs resulted in a decreased levels of aspartate aminotransferase and alanine aminotransferase through the modulation of the Notch1/HES1 and caveolin-1 signaling pathways in macrophages. The results of this study indicate that EV-mimic SPIONs have the ability to impede the movement of neutrophils and suppress inflammatory reactions, while avoiding any harmful effects on the liver. These findings suggest that EV-mimic SPIONs hold great potential as an effective anti-inflammatory treatment for liver injury in sepsis.

By mimicking the cargo protection potential of bEVs, scientists are now studying and developing mimics that have the ability to hold and protect therapeutic agents so as to release them at their target site. Zhang and colleagues (176) developed a copolymer carrier that is sensitive to enzymes produced by bacteria and have specific pH properties. The nanoparticles were layered with ICAM-1 antibodies after being loaded with 2-[(aminocarbonyl)amino]-5-(4-fluorophenyl)-3-thiophenecarboxamide) and ciprofloxacin. The findings of the study revealed that the nanoparticles were able to evade destruction at non-targeted sites and upon interaction with the vascular system of the infected liver tissue, the antibody-coated nanoparticles induced a decrease change in pH and enzymatic alterations, leading to the release of drugs (177, 178). These results suggest that the development of a drug-delivery system that mimics EVs could effectively target inflamed liver tissue and hold significant potential for the treatment of sepsis-associated liver injury.

Wang et al. (179) designed a biomimetic nanosystem with the purpose of facilitating early diagnosis of sepsis and for extracorporeal blood purification as a means of eliminating bacteria responsible for sepsis. This platform mimicked the structure, stability and biocompatibility properties of bEVs. The platform utilized iron oxide (Fe_3_O_4_) nanoparticles that were functionalized with chlorin e6 (Ce6) and bacteria-specific aptamers (Apt) (179). To mimic the stability and biocompatibility potential of bEVs, polyethylene glycol (PEG) molecules were introduced onto the magnetic nanoparticles. The utilization of the Apt facilitated the capture of bacteria, whereas the Fe_3_O_4_ NPs enabled magnetic separation for the purpose of detecting and enriching bacteria. Additionally, Ce6 functioned as a photosensitizer, demonstrating a notable degree of photosensitizing efficacy. The Fe_3_O_4_–Ce6–Apt nanosystem demonstrated efficacy in rapid diagnosis of sepsis resulting from the presence of one or more bacterial species, specifically *S. aureus* and *E. coli* (179). The Fe_3_O_4_–Ce6–Apt nanosystem was investigated for its potential in disinfecting contaminated blood samples. This was achieved by subjecting the samples to near-infrared (NIR) laser irradiation, which induced a photodynamic effect leading to bacterial death. Subsequently, the pathogens were eliminated through magnetic removal (179). The findings of this study indicate that the Fe_3_O_4_–Ce6–Apt nanosystem shows promise for sepsis treatment, as it allows for the safe reuse of disinfected blood for mice transfusion without any negative reactions (179).

The targeting ability, biocompatibility, and stability of bEVs have been mimicked to develop various biomimetics for biomedical purposes. Yu and colleagues developed and synthesized a nanosystem with high target to mitochondria to help scavenge excessive reactive oxygen species (ROS). Atorvastatin and mitochondria-targeting ceria (CeO_2_) NPs were combined in this nanosystem and used for the treatment of sepsis-induced acute kidney injury (180). To make this nanosystem responsive to ROS, mPEG-TK-PLGA polymer was coated on the surface of triphenylphosphine-functionalized CeO_2_ NPs. This enhanced the biocompatibility of the nanosystem. The resulting nanosystem was then loaded with atorvastatin, mimicking the protective function of bEVs in maintaining stability of their cargos (180). The accumulation of these NPs in the kidneys resulted in a specific target to the mitochondria leading to a reduction in the amount of expressed ROS levels. Additionally, the NPs showed antiapoptotic and antioxidant properties and effectively reduced inflammation (180).

Li et al. fabricated exosome mimics by incorporating various ratios of microRNAs that were upregulated by LPS into hyaluronic acid-polyethylenimine (HA-PEI) nanoparticles. The study demonstrated that the exosome mimics exhibited protective effects against sepsis in both murine and simian models (181).

In addition, alkaline solution, sonication, and buoyant density gradient ultracentrifugation were used to synthesized EV-mimetic ghost nanovesicles (EVs with potentially unwanted luminal cargos, such as cytosolic proteins and nucleic acids) loaded with dexamethasone. The ghost nanovesicles exhibited similar physical characteristics to endogenously released EVs, yet demonstrated a significantly greater production yield, approximately 200 times higher than that of EVs. The dexamethasone-loaded ghost nanovesicles exhibited a preserved topology similar to that of the parental cells. This characteristic led to a decrease in the release of IL-8 from endothelial cells treated with OMVs in an *in vitro* setting. Additionally, these nanovesicles demonstrated the ability to alleviate the symptoms associated with OMV-induced systemic inflammatory response syndrome (SIRS) in an *in vivo* model (182).

### 4.2 Cell-membrane-coated biomimetic nanoparticles for the treatment and prevention of bEV-induced sepsis

**Figure 4:**
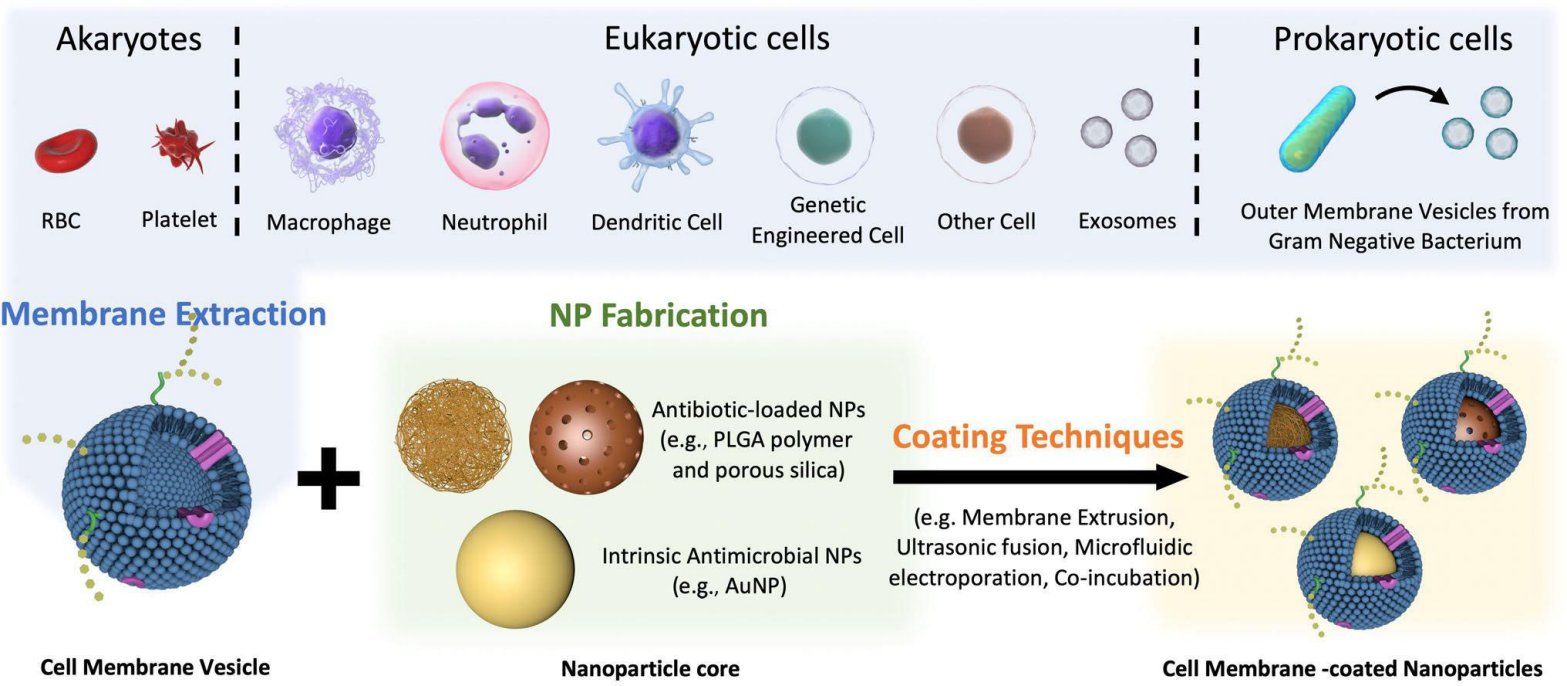
Schematic preparation of CMCNP. Several methods are employed to isolate cell membrane vesicles, including repeated freeze-thaw cycles, hypotonic lysis, ultrasonic disruption, and gradient centrifugation. For optimal stability, the core of cell membrane vesicles is enveloped with specially designed antibiotic-loaded NPs or intrinsic antimicrobial NPs. To successfully prepare CMCNP, a range of techniques are utilized to ensure the thorough encapsulation of cell membrane vesicles around the NP. These techniques include membrane extrusion, ultrasonic fusion, microfluidic electroporation, and co-incubation. Reproduced with permission (183). Copyright 2022, Wiley.

Cell membrane-coated nanoparticles (CMCNPs) are a type of biomimetic material that can be synthesized by enveloping cell membrane vesicles onto the surface of nanoparticles (NPs) using physical methods (183). In recent years, CMCNP, produced by combining the biological functions of cell membrane vesicles and the superior physicochemical properties of nanoparticles (NPs) has been used for various applications such as targeting infections, neutralizing EVs released by bacteria, and designing vaccines against bacterial infections. At present, there exist multiple techniques to synthesis CMCNPs. Membrane extraction, core nanoparticle synthesis, and fusing are the three distinct procedures to produce CMCNPs (**Figure 4**). The functionalization of the resultant nanoparticles depends on these processes.

**Figure 5:**
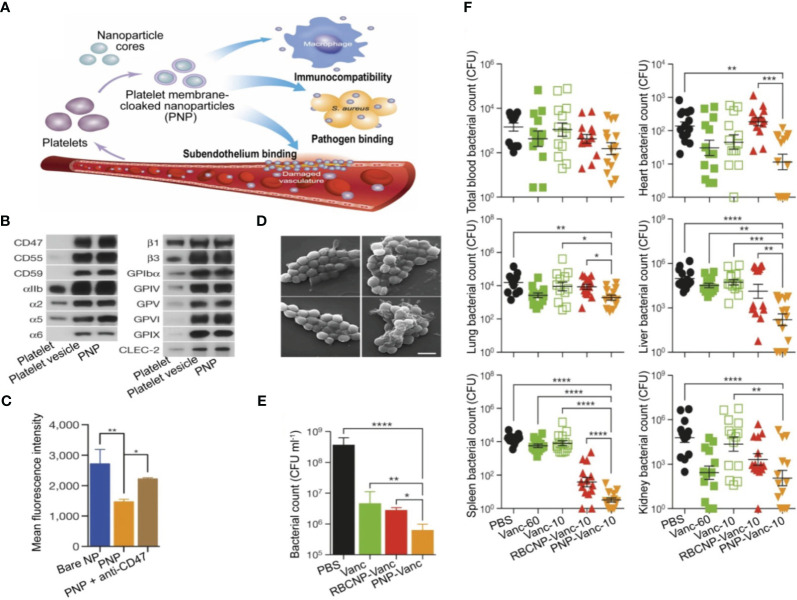
Utilization of PNPs to actively target and eradicate bacteria in the treatment of sepsis. **(A)** Illustration depicting the structure, composition, and properties of PNPs. Quantitative analysis on selected PL membrane proteins using Western blotting **(B)**. Flow cytometric analysis of phagocytized nanoparticles by human THP-1 cells **(C)**. Scanning tunnel microscopy images of MRSA252 bacteria incubated with PBS (top left), uncoated nanoparticles (top right), RBCNPs (bottom left), and PNPs (bottom right) **(D)**. The *in vitro* antimicrobial efficacy of Van in various forms **(E)**. An *in vivo* assessment of the antimicrobial efficacy of Van measured by quantifying bacterial counts in different organs (blood, heart, lung, liver, spleen, and kidney) of mice that were infected with MRSA252. The mice were subjected to various forms and doses of Van for a period of 3 days **(F)**. *P ≤ 0.05, **P ≤ 0.01, ***P ≤ 0.001, ****P ≤ 0.0001. Reproduced with permission (185). Copyright 2015, Springer Nature.

Platelets have the ability to engage in interactions with bacteria and bEVs (184), thereby endowing them with the capability of actively targeting and detoxifying these entities. A novel NP composed of poly(lactic-co-glycolic acid) (PLGA) was coated with human platelet derived from plasma membranes. This composite was combined with vancomycin (referred to as PNP-Van) and subjected to analysis. The findings demonstrated that PNP-Van exhibited notably potent antimicrobial properties in the liver and spleen, while displaying comparable efficacy in the blood, heart, lung, and kidney (**Figure 5**) (185). A study focused on investigating methicillin-resistant *Staphylococcus aureus* (MRSA) and methicillin-sensitive *Staphylococcus aureus* (MSSA) infection by developing a drug delivery system utilizing red blood cell membrane (RBCM). Initially, RBCM was loaded with polylactic acid-glycolic acid copolymer (PLGA) and subsequently coated with tedizolid (TR-701). Through the sustained release of the drug and simultaneous absorption of bEVs, this system showcased a favorable treatment outcome (186). Also, in the context of infection treatment, Fe_3_O_4_ was coated with a red blood cell (RBC) membrane to form RBC@Fe_3_O_4_ nanoparticles. The RBC@Fe_3_O_4_ nanoparticles demonstrated the ability to function as nano-sponge absorbent, effectively capturing EVs and toxins originating from MRSA. Subsequently, these captured entities were eradicated through the application of a photothermal process (187).

**Figure 6:**
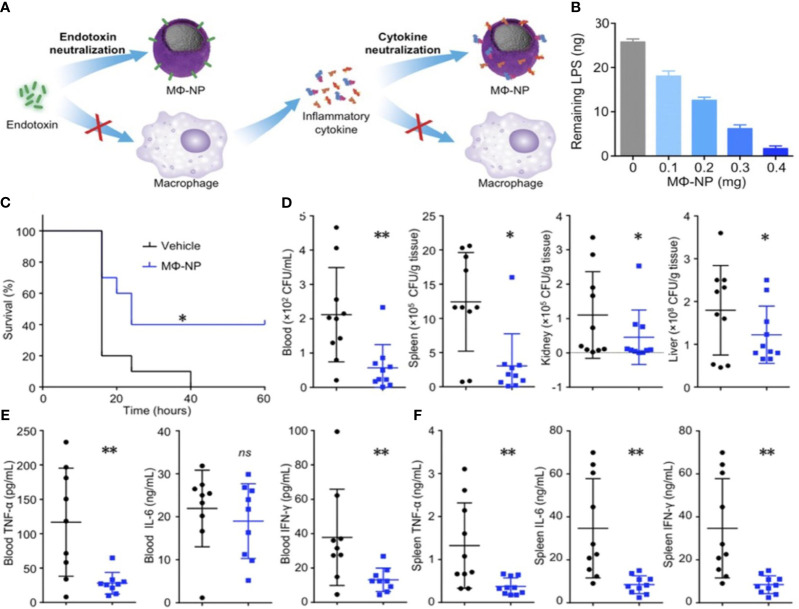
**(A)** This figure illustrates the processes of neutralizing endotoxin and inhibiting proinflammatory cytokines, which are two crucial mechanisms employed by MΦ-NPs in the treatment of sepsis. To measure the neutralization of LPS by MΦ-NPs *in vitro*, a fixed quantity of MΦ-NPs (0.4 mg) was introduced alongside different quantities of LPS **(B)**. The therapeutic effects of MΦ-NPs were evaluated by administering lethal doses of *E. coli*-induced bacteremia **(C–F)**. The survival curves of mice with bacteremia after treatment with MΦ-NPs (n = 10) **(C)**. Bacterial counts in the blood, spleen, kidney, and liver within 4 hours after intraperitoneal injection of MΦ-NPs **(D)**. Levels of pro-inflammatory cytokines, including IL-6, TNF-α, and IFN-γ, in blood and spleen samples **(E, F)**. ns, not significant; *P < 0.05, **P < 0.01. Reproduced with permission from (189). Copyright 2017, National Academy of Sciences.

Based on the potential interaction between receptors on the membrane of macrophage and PAMP in bacteria, Wang et al. (188) used macrophage membrane receptors that has been pretreated with bacteria in a nanosystem to achieve bacteria-specific targeted delivery. The association between CD14 and TLR-4 receptors on macrophage membrane is linked to the binding of LPS and the subsequent release of inflammatory factors. Consequently, the utilization of NPs that have been coated with macrophage membrane can effectively sequester LPS and subsequently inhibit the immune response. Thamphiwatana and colleagues utilized macrophage membrane-coated poly(lactic-co-glycolic acid) nanoparticles (MΦ-NPs) to effectively treat sepsis (**Figure 6A**) (189). In the *in vitro* setting, MΦ-NPs demonstrated the ability to neutralize 62.5 ng of LPS per mg. Additionally, they exhibited a concentration-dependent suppression of certain pro-inflammatory cytokines. These findings suggest that MΦ-NPs possess significant anti-inflammatory properties (**Figure 6B**). In a lethal-dose sepsis model induced by *Escherichia coli*, the group treated with MΦ-NPs exhibited a statistically significant improvement in survival outcomes. Additionally, this group showed a notable reduction in bacterial load in both the bloodstream and spleen (**Figures 6C, D**). The administration of MΦ-NPs resulted in a decrease in the levels of inflammatory cytokines, such as IL-6, TNF-α, and IFN-γ, in both blood and spleen, as a result of a decrease in bacterial load within these organs (**Figures 6E, F**). In contrast to conventional endotoxin neutralizers, MΦ-NPs exhibit the ability to impede endotoxin and macrophage binding pathways. This effectively suppressed a series of detrimental hyperinflammatory responses that are associated with clinical toxicity. Consequently, this approach presents a “universal” mechanism for neutralizing endotoxins generated by diverse Gram-negative bacteria, while concurrently impeding the hyperinflammatory response pathways initiated by these endotoxins (189).

The utilization of neutrophil membranes in the management of inflammation has been justified due to the ability of neutrophils to engage in diverse inflammatory reactions and their accumulation at sites of inflammation during pathogen elimination. Neutrophil membranes were extracted from natural neutrophils in a manner that preserved their biological properties. These membranes were then utilized to coat nanoparticles loaded with sparfloxacin (SPX), resulting in the production of camouflaged neutrophils (190). In contrast to conventional nano-medicines, the neutrophil membrane-coated nanoparticles (NM-NP-SPX) exhibited a comparable targeting capacity, akin to the ability of neutrophils to accumulate at sites of inflammation during an inflammatory response. Furthermore, it was observed that NM-NP-SPX exhibited extended duration of circulation within the body, while also possessing the property of controlled-release (190). In an *in vivo* experiment, it was observed that the levels of bacteria, inflammatory cytokines, and inflammatory cells in the lungs of mice with pneumonia exhibited a significant decrease within the first 24 h following the administration of NM-NP-SPX. This finding suggests that NM-NP-SPX possesses the potential to substantially mitigate the mortality risk associated with inflammation in patients with sepsis (190). **Table 3** summarizes various biomimetics used in the prevention and treatment of sepsis caused by bEVs.

**Table 3:** Prophylactic and therapeutic roles of various biomimetics for sepsis.

Name of biomimetic	Description	Mode of action	Ref
MSC-derived NVs	Mesenchymal stromal cells (MSCs) produced by serial extrusions of cells	Attenuate cytokine storm induced by OMVs by upregulating the production of IL-10	(168)
SPIONs	Iron oxide encapsulation with polyglucose sorbitol carboxymethyl ether	Reduce infiltration of inflammatory cells and enhance IL-10-producing macrophages via Cav1-Notch1/HES1-mediated autophagy	(175)
Fe_3_O_4_–Ce6–Apt nanosystem	Fe_3_O_4_ nanoparticles functionalized with chlorin e6 (Ce6) and bacteria-specific aptamers (Apt)	Detecting and enriching bacteria/bEVs by magnetic separation and eliminating the captured bacteria through photodynamic effect	(179)
Atv/PTP-TCeria NPs	Ceria nanoparticles modified with triphenylphosphine (TCeria NPs), followed by coating with mPEG-TK-PLGA and loaded with atorvastatin	Target mitochondria to eliminate excessive ROS, decrease oxidative stress and inflammation.	(180)
iExo-HA-PEI	microRNAs that have been upregulated by LPS in exosomes were incorporated into hyaluronic acid-polyethylenimine (HA-PEI) nanoparticles	Alleviates immune hyperactivation and cytokine storms	(181)
GhostEV-dexamethasone	“EV-mimetic ghost nanovesicles loaded with dexamethasone”	“Reduce the release of interleukin-8 from OMV-treated endothelial cells, and mitigate the symptoms of OMV-induced SIRS”	(182)
RBC@Fe_3_O_4_	Red blood cell (RBC) membrane-coated Fe_3_O_4_ nanoparticles	Act as nano-sponges to effectively capture EVs and toxins originating from MRSA and then kill them with a photothermal effect.	(187)
PNP-Van	poly(lactic-co-glycolic acid) (PLGA) coated with human platelet and combined with vancomycin	“Exploit the inherent bacterial adherence mechanism to platelet for targeted antibiotics delivery”	(185)
MΦ-NPs	Macrophage membrane-coated poly(lactic-co-glycolic acid) nanoparticles	Concurrently bind LPS and mitigate the expression of pro-inflammatory cytokines	(189)
NM-NP-SPX	Neutrophil membrane coated on nanoparticles loaded with sparfloxacin	Disguised as neutrophils to escape from the arrest by the immune system to arrive at the site of inflammation to precisely release the drug	(190)

## 5 Challenges

The primary unmet challenges in this field revolve around three key factors: (i) the need for cost-effective and efficient production and separation methods for bEVs; (ii) the establishment of standardized analytical techniques and production protocols; and (iii) the development of safe and effective modification strategies to enhance bEV functionality while minimizing potential toxicity.

### 5.1 Cost

The importance of scalability cannot be overstated in order to maintain a cost-effective production process of bEVs. Despite the fact that bacteria can be cultivated in large quantities using large fermentation vessels, the current yield of bEVs remains insufficient to achieve cost-effectiveness in their mass production (191). Various culture systems are currently being studied in order to enhance the production of bEV (192). However, the composition of bEVs may vary depending on the specific culture conditions employed. It is noteworthy that bacteria that have been genetically modified to have a weakened cell envelope have demonstrated successful outcomes in enhancing the secretion of EVs (98, 99). Therefore, it is crucial to actively pursue the exploration and advancement of novel methodologies that can efficiently expand the production of bEVs while maintaining cost-effectiveness.

### 5.2 Safety

The primary challenge in implementing bEVs in clinical settings is ensuring their safety. The primary constituent of OMVs, specifically LPS, elicits both immune responses and reactogenicity. Furthermore, it should be noted that various constituents, including outer membrane proteins and lipoproteins, have the potential to elicit systemic inflammatory reactions. In light of the safety concerns surrounding the utilization of bEVs for drug delivery or as vaccines, there exist several strategies that can be employed to improve immunogenicity and mitigate potential toxic effects. (i) Genes involved in the synthesis of LPS, such as *msbA*, *lpxM*, *lpxL1*, or *msbB*, can be genetically modify in order to decrease the production of LPS (191). (ii) In order to selectively decrease the content of LPS, it is recommended to employ physical or chemical extraction methods for bEVs (193). (iii) To prevent the induction of systemic inflammation, OMVs can be enveloped with a pH-sensitive calcium phosphate (CaP) shell prior to their delivery to the intended site (194). Additionally, it is possible to enhance culture conditions in order to optimize the large-scale production of bioengineered vesicles (bEVs). In order to ensure consistent and replicable isolation of bEVs, it is imperative to establish standardized protocols that take into account their morphological, biochemical composition, and biophysical properties. Moreover, in order to facilitate the clinical translation of bEVs, it is imperative to advance the development of economically viable methodologies for their isolation and purification. To create quick and sensitive detection platforms for diagnostic applications, it is critical to miniaturize bEV separation technologies that enable effective and resilient isolation from limited biological samples.

Regrettably, outer membrane vesicles (OMVs) that are defficient in LPS demonstrate a diminished level of immunogenicity in comparison to OMVs that possess normal levels of LPS (195). As a result, a novel task emerges in the quest to find ultimate balance between minimal toxicity and maximal immunogenicity. In order to achieve this objective, it is imperative to conduct high-throughput screenings to investigate the potential synergistic impact of various pattern recognition receptor agonists. By employing this approach, researchers can identify the optimal combination of adjuvants (196). Moreover, it is crucial to conduct thorough safety evaluations for bEV-based drugs, which should encompass pharmacokinetic/toxicokinetic studies, absorption, metabolism, excretion tests, and distribution. These assessments are essential in order to gain a comprehensive understanding of the toxicity associated with such drugs.

### 5.3 Standardization and reproducibility

Research on bEVs continues to present challenges due to the absence of standardized preparatory and analytical techniques. The variability and integrity of isolated extracellular vesicle populations, resulting from diverse production and isolation methods, pose challenges to the consistency and replicability of findings across different studies (192, 197). Furthermore, it should be noted that the various techniques employed for the quantification of bEV protein and its quantity have the potential to influence experimental results (198). Regrettably, the persistence of these issues can be attributed to the current absence of adequate methodologies and standardized protocols for conducting comprehensive studies on bEVs. In order to facilitate comprehensive research and effective clinical application, there is an urgent need for the establishment of standardized guidelines. These guidelines should be similar to the ones currently available for studies involving host-derived extracellular vesicles (199).

## 6 Conclusion and future perspective

Higher biocompatibility and reduced malignancy risks of bEVs render them more advantageous compared to their parent bacteria. Moreover, the intricate composition of the bioactive molecules, coupled with their ability to traverse cellular barriers and penetrate various tissues, renders them highly suitable for a wide range of biomedical applications. We posit that conducting mechanistic investigations on the systemic activity of bEVs is imperative in order to comprehensively understand their influence in sepsis. Furthermore, such studies will aid in the identification of potential therapeutic targets, thereby facilitating the development of effective treatment strategies for sepsis and sepsis-associated diseases. The utilization of orthogonal separation techniques for the fractionation of intricate matrices, such as blood, in conjunction with highly sensitive and microbe-specific detection methods, can play a crucial role in uncovering the concealed phase of bEVs-derived sepsis or sepsis-associated conditions. In order to ensure the safe clinical utilization of endotoxins, it is imperative to implement measures aimed at reducing their levels. In addition, comprehensive research is needed to eliminate endotoxins while maintaining bEV immunogenicity. It is essential to strike the right balance between low toxicity and high immunogenicity. In addition, based on a comprehensive understanding of its active components, the use of modified bEVs with detoxification controllable components can provide a reliable strategy for enhancing its immunogenicity and reducing its toxicity. Furthermore, although there have been varying results regarding the use of bEVs as diagnostic markers in biofluids, they possess significant potential in the field of disease diagnosis. However, there are still certain challenges that hinder its integration into clinical applications (28). Factors such as age, dietary habits, comorbidities, body mass index (BMI), etc, are uncontrolled confounding variables that have the potential to compromise the accuracy of utilizing bEVs as a means to define a pathological condition. Therefore, it is imperative for future research endeavors to account for these confounding variables in order to accurately assess the diagnostic efficacy of bEVs.

## Author contributions

CE: Conceptualization, Supervision, Writing – original draft, Writing – review & editing. XD: Conceptualization, Funding acquisition, Supervision, Writing – review & editing. ED: Writing – review & editing. XL: Writing – review & editing. RT: Writing – review & editing. TS: Conceptualization, Funding acquisition, Supervision, Writing – review & editing.
